# Reduced workforce participation 5 years prior to first Parkinson’s disease sick-leave

**DOI:** 10.1038/s41531-018-0072-2

**Published:** 2018-12-12

**Authors:** Jonathan Timpka, Örjan Dahlström, Armin Spreco, Maria H. Nilsson, Susanne Iwarsson, Toomas Timpka, Per Odin

**Affiliations:** 1 Department of Clinical Sciences Lund, Neurology, Lund University, Lund, Sweden; 2 Department of Neurology, Skåne University Hospital, Lund, Sweden; 3 Department of Behavioural Sciences and Learning, Linköping University, Linköping, Sweden; 4 Athletics Research Center, Linköping University, Linköping, Sweden; 5 Department of Medical and Health Sciences, Linköping University, Linköping, Sweden; 6 Centre for Healthcare Development, Region Östergötland, Linköping, Sweden; 7 Department of Health Sciences, Lund University, Lund, Sweden; 8 Memory Clinic, Skåne University Hospital, Malmö, Sweden; 9 Department of Neurology, Central Hospital, Bremerhaven, Germany

## Abstract

The importance of understanding the prodromal phase of Parkinson’s disease (PD) by systematic recording of prediagnostic symptoms and reductions in body functions has been highlighted. The aim of this study was to investigate whether persons later diagnosed with PD exhibit increased physician-certified sickness absence 1, 2, and 5 years prior to a first sick-leave episode attributed to PD. A case-control study was performed to analyze data from all nontrivial (exceeding 14 days) sick-leave episodes in Sweden between 2008 and 2014. The 537 incident PD sick-leave episodes were identified as PD sick-leave cases and compared to 537 sick-leave controls identified by matching age, sex, and date of the first day of the sick-leave episode. The total sickness absence and sickness absence due to musculoskeletal diagnoses were found to be increased among the PD sick-leave cases from 5 years prior to the first sick-leave episode ascribed to PD when compared to the controls. No differences between PD sick-leave cases and sick-leave controls were found with regard to mental and behavioral diagnoses. We conclude that the capacity to participate in working life is reduced already at the early prediagnostic stages of PD. This finding can be used as a basis for further research into the process of identifying individuals at risk for developing PD, particularly in combination with further investigation into biochemical, genetic, and imaging biomarkers.

## Early symptoms: Clues in work sickness absences

A Swedish study shows that Parkinson’s disease (PD) patients had more sick-leave episodes in the years before diagnosis than matched controls not diagnosed with PD. There is growing evidence that prior PD diagnosis, patients experience a variety of nonmotor symptoms and subtle motor signs. Further understanding these symptoms could prompt earlier therapeutic intervention and increase the likelihood of slowing down or even halting disease progression. A study led by Jonathan Timpka at Lund University examined the sickness leave history of 537 workers 1, 2 and 5 years before a first sick-leave episode due to PD. These workers took more sick-leave due to musculoskeletal symptoms, but not due to mental or behavioral issues, than the control group. These findings highlight a potential effect of preclinical PD on patients’ ability to work.

## Introduction

Many countries in Europe and North America experience demographic changes with ageing populations that within 20 years will lead to a 50% increase in the number of persons living with Parkinson’s disease (PD), and an even greater increase is predicted for developing countries.^1,2^ Nonmotor symptoms such as depression and anxiety are seen as part of the prodromal phase of PD and often arise at least 10–20 years prior to the onset of motor symptoms.^3^ Furthermore, there is a slightly increased risk for being diagnosed with PD during the 10 years following an injurious fall and the 26 years following a hip fracture.^4^ PD pathophysiology is currently recognized to comprise progressive destruction of several brain regions, from the brain stem and the basic forebrain to the extrapyramidal system,^5–7^ and the importance of a more thorough understanding of the prodromal phase by recording symptoms and clinical findings reflecting this prediagnostic process of PD has been highlighted.^8^ At least 30% of people presently living with PD are of working age.^9^ At the group level, persons who later will get diagnosed with PD have been reported to exhibit higher medical expenses and a lower employment rate up to 8 years prior to the diagnosis in comparison with controls.^10^ However, the patterns through which early nonspecific motor and nonmotor symptoms affect workforce participation during prodromal and early PD have not been systematically investigated.

There is a largely unexplored opportunity for a better understanding of the effects of early and prodromal PD by detailed studies of the prediagnostic capacity to participate in working life. This study aimed to investigate whether persons diagnosed with PD exhibit increased sickness absence (for all diagnoses, as well as for mental and behavioral or musculoskeletal diagnoses, respectively) 1, 2, and 5 years prior to a first sick-leave episode attributed to PD. A secondary purpose was to gain knowledge about the possibility to identify individuals at risk of developing PD in order to allow diagnosis and treatment before motor symptoms occur.

## Results

**Fig. 1:**
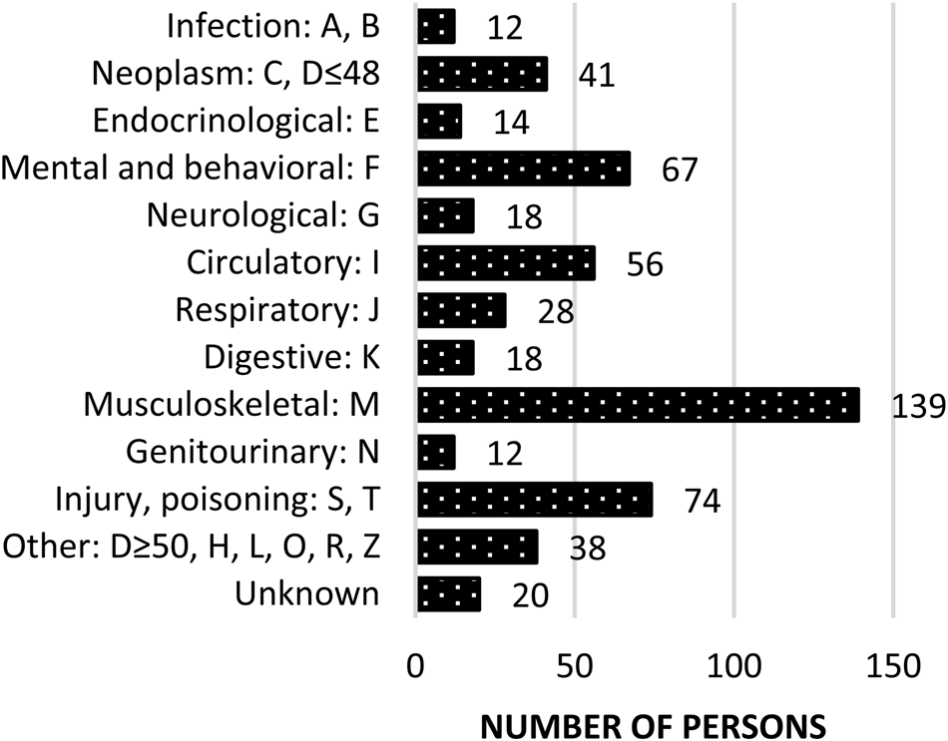
Distribution of ICD-10 diagnoses in the control group (*n* = 537) for the sick-leave episode used for matching with incident Parkinson’s disease sick-leave cases. ICD-10 Tenth revision of International Classification of Diseases and Related Health Problems

A total of 537 incident PD sick-leave cases and 537 sick-leave controls with other diagnoses were identified (Table 1, Fig. 1). A majority of the PD sick-leave cases were men (63.7%) and the median age was 59 years.

**Table 1:** Demographic characteristics of the persons with Parkinson’s disease (PD) and controls

	PD	Control	Total
*N*	537	537	1074
Age, years, median (min−max)	59 (21–67)	59 (21–67)	59 (21–67)
Sex, % male/female	63.7/36.3	63.7/36.3	63.7/36.3
Income in USD, mean (SD)	35,360 (18,800)	35,391 (14,591)	35,376 (16,820)
Occupation according to SSYK-96, *n* (%)			
1. Managers	25 (5.7)	24 (5.9)	49 (5.8)
2. Occupation requiring advanced level of higher education	91 (20.7)	66 (16.2)	157 (18.5)
3. Occupation requiring higher education or equivalent	94 (21.4)	61 (15.0)	155 (18.3)
4. Administration and customer service	29 (6.6)	34 (8.4)	63 (7.4)
5. Service, care and shop sales	76 (17.3)	75 (18.4)	151 (17.8)
6. Agri-, horticultural, forestry, fishery	14 (3.2)	5 (1.2)	19 (2.2)
7. Building and manufacturing	43 (9.8)	58 (14.3)	101 (11.9)
8. Mechanical manufacturing, transport, etc.	42 (9.5)	60 (14.7)	102 (12.0)
9. Elementary occupations	25 (5.7)	23 (5.7)	48 (5.7)
10. Armed forces	1 (0.2)	1 (0.2)	2 (0.2)
Unknown	97	130	227
Employment situation			
Employed	453 (85.8)	470 (89.5)	923 (87.7)
Unemployed	43 (8.1)	32 (6.1)	75 (7.1)
Self-employed	32 (6.1)	23 (4.4)	55 (5.2)
Unknown	9	12	21

Persons with an incident PD sick-leave during 2008–2014 (*n* = 537) and a sick-leave control group (*n* = 537) matched for sex, age, and date of sick-leave. Incomes were converted to USD from SEK (1:8.43)

*USD* U.S. dollar, *SSYK-96* Swedish Standard Classification of Occupations 1996, *SEK* Swedish krona

### Employment and occupational activity

Fitting the occupational and employment data into a multiple logistic regression model with case status as outcome variable showed that PD sick-leave cases were less likely to work in occupations with lower education requirements in comparison to sick-leave controls (odds ratio (OR) 0.61, 95% confidence interval (CI) 0.46–0.80, *P* < .001). PD sick-leave cases were also more likely to be self-employed or unemployed in comparison to sick-leave controls (OR 2.16, 95% CI 1.28–3.62, *P* *=* .004).

### Sickness absence prevalence

Using McNemar’s test, a larger portion of PD sick-leave cases than sick-leave controls were found to have had ≥1 sick-leave episode 1, 2, and 5 years prior to the incident PD sick-leave (*P* < .001 for each of the three time spans; Table 2). Moreover, a larger portion of PD sick-leave cases had ≥1 sick-leave episode due to one or more musculoskeletal diagnoses 1 year (*P* = .001), 2 years (*P* = .036), and 5 years (*P* = .006) prior to the incident PD sick-leave compared to the sick-leave controls. No significant differences in the portion of persons with ≥1 sick-leave episode due to mental and behavioral diagnoses were seen between PD sick-leave cases and sick-leave controls neither 1, 2, nor 5 years prior to the incident PD sick-leave.

**Table 2:** Prevalence of ≥1 sick-leave episode among persons with an incident Parkinson’s disease (PD) sick-leave and controls sick-listed at the same date for other diagnoses

	Total	PD	Control	*P*	OR (95% CI)
*N*	1074	537	537		
≥1 sick-leave episode reported, *n* (%)					
One year prior to incident sick-leave					
Any diagnosis	159 (14.8)	121 (22.5)	38 (7.1)	<.001	3.8 (2.6–5.6)
Musculoskeletal diagnosis	57 (5.3)	41 (7.6)	16 (3.0)	.001	2.7 (1.5–4.9)
Mental and behavioral diagnosis	20 (1.9)	14 (2.6)	6 (1.1)	.096	2.4 (0.9–6.2)
Two years prior to incident sick-leave					
Any diagnosis	247 (23.0)	164 (30.5)	83 (15.5)	<.001	2.4 (1.8–3.2)
Musculoskeletal diagnosis	86 (8.0)	53 (9.9)	33 (6.1)	.036	1.7 (1.1–2.6)
Mental and behavioral diagnosis	36 (3.4)	22 (4.1)	14 (2.6)	.230	1.6 (0.8–3.2)
Five years prior to incident sick-leave					
Any diagnosis	432 (40.2)	249 (46.4)	183 (34.1)	<.001	1.7 (1.3–2.1)
Musculoskeletal diagnosis	146 (13.6)	89 (16.6)	57 (10.6)	.006	1.7 (1.2–2.4)
Mental and behavioral diagnosis	95 (8.8)	46 (8.6)	49 (9.1)	.826	0.9 (0.6–1.4)

Number and portion of incident PD sick-leave cases (*n* = 537) and sick-leave controls (matched for sex, age, and date of sick-leave; *n* = 537) with ≥1 sick-leave episode in the 1-, 2-, and 5-year-period preceding the sick-leave used for matching. McNemar’s test was used for comparisons. The tenth revision of International Classification of Diseases and Related Health Problems (ICD-10) was used for classification of diagnoses

*OR* odds ratio, *CI* confidence interval

### Sickness absence quantity

Using Wilcoxon signed-rank test, PD sick-leave cases were found to have had a higher number of total days on sick-leave than sick-leave controls 1 year (*P* < .001), 2 years (*P* < .001), and 5 years (*P* = .001) prior to the incident PD sick-leave (Table 3). The number of sick-leave days due to musculoskeletal diagnoses was significantly higher among PD sick-leave cases than sick-leave controls 1 year (*P* *=* .009), 2 years (*P* = .023), and 5 years (*P* = .033) prior to the incident PD sick-leave. For mental and behavioral diagnoses, no significant differences were found between PD sick-leave cases and sick-leave controls regarding the number of days spent on sick-leave during either of the three studied time spans.

**Table 3:** Number of historical sick-leave days among persons with an incident Parkinson’s disease (PD) sick-leave and controls sick-listed at the same date for other diagnoses (all medians = 0)

Sick-leave days	PD (*n* = 537) Percentiles					Control (*n* = 537) Percentiles						
	75	90	95	97.5	Max	75	90	95	97.5	Max	*P*	*r*
One year prior to incident sick-leave												
Any diagnosis	0	85	210	275	339	0	0	43	272	361	<.001	.16
Musculoskeletal diagnosis	0	0	51	166	280	0	0	0	30	361	.009	.08
Mental and behavioral diagnosis	0	0	0	30	339	0	0	0	0	324	.085	
Two years prior to incident sick-leave												
Any diagnosis	26	190	456	640	704	0	62	194	383	687	<.001	.14
Musculoskeletal diagnosis	0	3	101	303	641	0	0	28	109	499	.023	.07
Mental and behavioral diagnosis	0	0	0	91	704	0	0	0	27	677	.228	
Five years prior to incident sick-leave												
Any diagnosis	141	617	975	1185	1668	62	421	741	1140	1737	.001	.10
Musculoskeletal diagnosis	0	80	270	874	1668	0	22	149	500	1595	.033	.07
Mental and behavioral diagnosis	0	0	90	306	1403	0	0	147	540	1615	.804	

Distribution of historical sick-leave days among incident PD sick-leave cases (*n* = 537) and sick-leave controls (matched for sex, age, and date of sick-leave; *n* = 537) in the 1-, 2-, and 5-year-period preceding the sick-leave used for matching. Sick-leave episodes ≤14 days and days 1–14 of sick-leave episodes >14 days are not shown. Wilcoxon signed-rank test was used for comparisons. The tenth revision of International Classification of Diseases and Related Health Problems (ICD-10) was used for classification of diagnoses

## Discussion

The primary finding from this study is that persons who later were allowed sick-leave due to PD were more absent from work due to illness than matched sick-leave controls already 5 years prior to the incident PD sick-leave episode. We found specifically that persons later diagnosed with PD had been more absent from work with reference to musculoskeletal diagnoses 1, 2, and 5 years prior to the incident PD sick-leave episode, while there was no increase in sickness absence with regards to mental and behavioral diagnoses.

Regarding the finding that persons with PD exhibit increased prediagnosis sickness absence due to musculoskeletal disorders, a recent study from secondary care in Denmark reported that musculoskeletal diagnoses in general—and lumbar pain in particular—are more common among patients that 3 years later receive a PD diagnosis than among those that do not.^11^ Similar findings based on data from primary care have also been presented.^12^ Pain is a nonmotor symptom that is commonly reported to appear in the prodromal phase of PD.^12–14^ It is possible that an increased occurrence of pain is a partial explanation of the increased sickness absence due to musculoskeletal diagnoses in the present study. Furthermore, the occurrence of tremor, fatigue, dizziness, shoulder pain or stiffness, balance impairments, rigidity, and hypotension are overrepresented in persons 2−10 years prior to a PD diagnosis;^12^ factors that either on their own or indirectly could result in sickness absence due to musculoskeletal diagnoses.

There were no differences regarding mental and behavioral diagnoses between the PD sick-leave cases and sick-leave controls in the present study. However, in a previous small cross-sectional study of working-age persons with PD, we found anxiety to be associated with early retirement and sickness absence.^15^ Anxiety, together with depression and anhedonia, are known to be common from an early stage of the disease process and are all among the nonmotor symptoms that are regarded as part of the prodromal phase of PD.^3,14,16,17^ There are several possible explanations to the discrepancies between the present and previous studies. Comorbidity with musculoskeletal and mental health issues are common in people with PD and it is possible that physicians chose to use the less stigmatizing—i.e. musculoskeletal—diagnosis on the sickness certification form when confronted with both somatic and psychiatric illness. Our study also included persons who had received a sickness certification from a physician at any level of the health-care system and did not only involve persons diagnosed in hospital care.^11^ This difference in the selection of the study populations could contribute to the discrepancies between the findings. Research on depression and mental health issues in PD is further complicated by the fact that neither PD duration, stage, severity, or age of onset is consistently associated with the occurrence or severity of depressive episodes in PD.^18^ However, the retrospective design of this study does not permit drawing conclusions about causal mechanisms, implying that more research on comorbidity between mental and behavioral disease and PD is warranted.

We also noted that individuals in the PD group were less likely to be involved in manual work (Table 1). It is possible that this difference is caused by a gradual increase of symptoms, which influences the selection of occupation, or may be attributed to shared personality traits among persons with PD.^19–21^ Moreover, the interplay between symptom and type of occupation may also have influenced the likelihood of individuals getting sick-listed, due to that occupations vary with regard to whether a specific symptom becomes a limiting factor for executing work tasks. Furthermore, the observed tendency (post-hoc Chi-square, *P* = .055) towards that agricultural occupations were more common in the PD group corresponds with the notion that pesticide exposure could be a risk factor for PD;^22^ however, the design and size of this study does not allow for any conclusions regarding these occupational differences.

Further knowledge about factors useful for early identification of persons with a high risk for developing PD is needed. Soon, when one or more treatments for PD presumably have been proven as being disease modifying, there will be a great increase in the need for early and accurate diagnosis to improve long-term outcomes. Earlier studies on workforce participation in PD point to several important challenges. After diagnosis, persons with PD have been reported to register more sick days than age- and sex-matched controls and seldom continue to work full-time for more than a few years.^23–25^ The majority of those who are diagnosed with PD whilst of working age leave the workforce within 5–10 years,^23,25^ i.e., 4–7 years earlier than the general population.^23,26,27^ By using retrospective workforce participation data preceding an incident PD sick-leave, this study instead contributes to a better understanding of what is likely to be clinically relevant early symptoms related to PD. Based on our findings, screening for musculoskeletal symptoms could have a place besides screening for combinations of nonmotor symptoms such as olfactory impairment, rapid eye movement sleep behavior disorder, and autonomic dysfunction.^8,28^ However, this is likely to have to be combined with biochemical, genetic, and imaging biomarkers to be both sensitive and specific enough.^29^

There are several study limitations deserving consideration. In Sweden, the employer compensates the employee during the first 14 days of a sick-leave episode, and a physician’s certificate with a diagnosis is only required after day 7. Thus, reliable diagnoses for sick-leave episodes lasting 14 days or less are not systematically available and were therefore not included in the analyses. This means that common, but more trivial, short sick-leave episodes due to for example infections or less severe reactions to stress are not included in this study. Furthermore, the control group was likely to have more health problems than the general population, which could lead to a relative underestimation of the illness burden in the PD group compared to the general population. Lastly, we have no data on the dates of PD diagnosis in the study sample and no information beyond the clinical experience of how the date of diagnosis relates to the first sick-leave episode attributed to PD.

The results of the present study suggest that nontrivial sickness absence is increased among persons with PD when compared to controls at least 5 years prior to the first sick-leave episode ascribed to PD, both in terms of total sickness absence and in sickness absence due to musculoskeletal diagnoses. No specific increase was detected for mental and behavioral diagnoses. The studying of sick-leave history among persons with PD is an opportunity to increase the knowledge about the disease process per se. The screening of early nonspecific motor symptoms may also be of future use, together with other forms of biomarker screening, to identify persons with prodromal PD. Although intervention during the prodromal stage of PD is currently unfeasible, our results point to the need for interventions addressing workforce participation issues soon after diagnosis as the decline in work ability may have started several years earlier.

## Methods

### Data sources

For this register-based study, we used a retrospective case-control design for analyses of the “Support for Righteous Sick-leave” database (SRS), which contains data on all persons in Sweden having been compensated through the national sickness insurance for a sick-leave episode lasting longer than 14 days between years 2008 and 2014. Sick-leave episodes lasting 14 days or shorter are in Sweden compensated by the employer and are not included in this study. For the included persons and sick-leave episodes, SRS contains data on all previous sickness absence between years 1994 and 2007, the diagnoses stated in the sickness certification, employment details, education, and income. The sickness certification diagnoses are coded using the tenth revision of the International Classification of Diseases and Related Health Problems (ICD-10) at the three-character level. Occupations are categorized in accordance with the Swedish Standard Classification of Occupations (SSYK) 1996. The SRS database is compiled from the Swedish Social Insurance Agency’s registry Micro-Data for Analysis of the Social Insurance (MiDAS) and the version used for this study covered 7.8 million sick-leave episodes.

### Ethical approval

The study was approved by the Regional Ethics Board in Linköping (dnr. 2014/462–31) with waived informed consent due to the retrospective nature of the study using publicly available information.

### Statistical analysis

Persons with a first sick-leave episode attributed to PD (incident PD sick-leave cases) were matched by age (exact years), sex, and date of sick-leave to controls with non-PD diagnoses at a 1:1 ratio. An incident PD sick-leave case was defined as an individual with a first sick-leave episode that was exceeding 14 days and was based on the ICD-10 diagnosis code for PD (G20), while a non-PD sick-leave control was an individual with a sick-leave episode that was exceeding 14 days and was based on any other diagnosis than PD.

The ten different SSYK categories were dichotomized in two ways: first by education (higher education: categories 1–3; lower education: categories 4–9 and 0), then agricultural occupations or not (agricultural, horticultural, forestry, or fishery: category 6; other occupations: 0–5 and 7–9). Differences regarding occupation and employment status between PD sick-leave cases and sick-leave controls at the time of selection were analyzed by fitting occupation and employment data into a multiple logistic regression model. PD sick-leave case/sick-leave control (1/0) was used as the response variable and occupation and employment status as explanatory variables. A chi-square test was used for post-hoc testing of differences in the prevalence of agricultural occupations between PD sick-leave cases and sick-leave controls.

Paired comparisons between PD sick-leave cases and sick-leave controls of the prevalence of sick-leave episodes exceeding 14 days 1, 2, and 5 years prior to the incident PD sick-leave were made with McNemar’s test for dichotomous data. The dichotomizations were made based on whether the person had ≥1 sick-leave episode or not. For comparisons between PD sick-leave cases and sick-leave controls of the cumulative number of sick-leave days, the Wilcoxon signed-rank test was used.

*P* < .050 was considered statistically significant.

### Code availability

IBM SPSS Statistics for Windows (version 24.0) was used for statistical analyses. No custom code was generated for the study.

## Data Availability

No additional data can be provided by the authors. Data from the MiDAS database are available through request from the Swedish Social Insurance Agency.
